# Pentamidine inhibits proliferation, migration and invasion in endometrial cancer via the PI3K/AKT signaling pathway

**DOI:** 10.1186/s12905-022-02078-1

**Published:** 2022-11-24

**Authors:** Lin Lin, Yunan Gao, Xiaochen Hu, Jiabao Ouyang, Chunbo Liu

**Affiliations:** 1 Department of Nuclear Medicine, The First Affiliated Hospital of Harbin Medical University, Harbin City, 150001 Heilongjiang Province People’s Republic of China; 2 Department of Cardiology, The Fourth Affiliated Hospital of Harbin Medical University, Harbin City, 150001 Heilongjiang Province People’s Republic of China; 3 Department of Respiratory Medicine, The First Affiliated Hospital of Harbin Medical University, Harbin city, 150001 Heilongjiang Province People’s Republic of China; 4 Ultrasound Department, The First Affiliated Hospital of Harbin Medical University, Harbin city, 150001 Heilongjiang Province People’s Republic of China

**Keywords:** Pentamidine, Endometrial cancer, PI3K/AKT pathway, LY294002

## Abstract

**Background:**

Pentamidine has been reported to have many pharmacological effects including anti- protozoal, anti-inflammatory, and anti-tumor activities. The aim of this study is to investigate the potential therapeutic role of Pentamidine and molecular mechanisms of Pentamidine on PI3K/AKT signaling pathway underlying the anti-tumor properties in endometrial cancer.

**Methods:**

Our study was carried out in the central laboratory of Harbin Medical University from 2019 to 2021. Human endometrial cancer cell lines Ishikawa and HEC-1A were treated with Pentamidine. The proliferation ability of cells was investigated by MTS and colony formation assays. The cell cycle distribution was detected by flow cytometry. Cell migration and invasion were analyzed by using the wound healing assay and Transwell assay. Western blotting was performed to measure the levels of AKT, p-AKT, MMP-2, and MMP-9.

**Results:**

Our results revealed that treatment of Pentamidine inhibited proliferation, migration and invasion of Ishikawa and HEC-1A endometrial cancer cells. Mechanistic investigation showed that Pentamidine inhibited PI3K/AKT signaling pathway and also reduced the expression of MMP-2 and MMP-9. In addition, co-treatment with PI3K kinase inhibitor LY294002 and Pentamidine leaded to increased repression of cell viability and the protein expression of p-AKT in Ishikawa cells.

**Conclusions:**

Pentamidine suppresses PI3K/AKT signaling pathway, and inhibits proliferation, migration and invasion of EC cells. These findings suggested that Pentamidine might be a potential candidate for treating EC through PI3K/AKT pathway.

**Supplementary Information:**

The online version contains supplementary material available at 10.1186/s12905-022-02078-1.

## Background

Endometrial cancer (EC), a malignant epithelial tumor in lining of the uterus, is the most common malignancy of the female genital tract [1]. Endometrial cancer frequently occurs in post-menopausal women, and the endometrium is a well-known site of cancer affecting women [2]. Recent years, there has been an increase in the incidence of EC for both pre- and post-menopausal women. However, minimal progress has been made to improve the 5-year overall survival for women diagnosed with endometrial cancer in the past decades [3]. Therefore, it is necessary to search for novel treatment strategies for improving current therapeutic landscape of EC.

Globally, endometrial cancer ranks as the sixth most common cancer in women and is the 14th leading cause of cancer related death [3, 4]. Endometrial cancer is the most common gynecological cancer in industrialized countries and second, after cervical cancer, in the developing world including India and China [5]. In 2018, around 382,100 new cases of endometrial cancer were reported worldwide, accounting for about 4.4% of all new cases of cancer in women [6]. The morbidity and mortality of EC are annually increasing, which severely threatens woman's health. As EC is diagnosed at early stages, it presents a relatively good prognosis. However, the prognosis of women with advanced or recurrent endometrial cancer is poor with a median overall survival (OS) of approximately 7–10 months [7]. Currently, options for treatment of advanced or recurrent disease remain limited and choices were limited to chemotherapy [8, 9]. In advanced and recurrent EC, there remains a high unmet need, highlighted by the fact that within the last 3 decades there have only been a few drugs approved for use [10]. Therefore, novel effective anti-cancer drugs are urgently needed for inhibiting the malignant progression of EC.

Pentamidine is an aromatic diamine drug that has been used in the treatment of human protozoa infections for many decades [11]. Pentamidine is used as an agent for treating African trypanosomiasis, antimony resistant leishmaniasis and Pneumocystis carinii pneumonia [11–14]. More recently, several research groups have demonstrated the anti-cancer effects of Pentamidine in human cancer cell lines (e.g., melanoma, lung, ovarian, colon, prostate and breast cancers), mice models of melanoma and clinical trials [15–20]. It has been reported that Pentamidine suppresses the expression of hypoxia-inducible factor 1 alpha (HIF-1a) which correlates with increased vascularity, resistance to chemotherapy and radiotherapy, and poor prognosis in DU145 prostate and MDA-MB-231 breast cancer cell lines [11]. Moreover, Pentamidine inhibits prostate cancer progression via selectively inducing mitochondrial DNA depletion and dysfunction [21]. However, the effects and the mechanisms of Pentamidine on EC have not been explored to date.

The phosphatidylinositol 3-kinase (PI3K)/protein kinase B (AKT) signaling pathway is extensively presented in different types of cells and involved in the regulation of multiple cellular physiological processes by activating downstream corresponding effector molecules, which serve an important role in the cell cycle, growth, proliferation and differentiation [22, 23]. Abnormal activation of P13K/AKT can lead to infinite cell proliferation and resistance to apoptosis, which is associated with the occurrence of many tumors [22]. A number of studies suggest that the PI3K/AKT signaling pathway is associated with certain gynecological tumors, including endometrial cancer [22, 24]. The PI3K/AKT signaling pathway is a frequently altered signaling pathway in endometrial cancer [22]. Furthermore, excessive activation of PI3K/AKT may produce drug resistance in tumors, including endometrial cancer [22, 24]. Therefore, developing new drug targets for PI3K/AKT signaling pathway may prove beneficial to prevent progression of EC.

In the present study, we identified Pentamidine’s ability to inhibit proliferation, migration and invasion as well as induce a block in G1/S progression in EC cells. Further, we demonstrated that Pentamidine reduces phosphorylation levels of AKT and inhibits the expression of MMP-2/9 through PI3K/AKT pathway in EC cells. Moreover, Pentamidine enhances inhibitory role of PI3K kinase inhibitor LY294002 in cell proliferation and the expression of phosphorylated (p)-AKT in Ishikawa EC cell lines. Taken together, our results suggested that Pentamidine represses PI3K/AKT/MMP-2/9 signaling pathway resulting in inhibition of proliferation, migration and invasion of EC cells.

## Materials and methods

### Chemicals

Pentamidine and LY294002 were obtained from MedChemExpress (MCE, HY-B0537B, HY-10108), and were dissolved in DMSO.

### Cell lines and cell culture

Endometrial cancer cells Ishikawa (highly differentiated endometrial cancer) and HEC-1A (moderately differentiated endometrial cancer) were purchased from American Type Culture Collection (ATCC). Ishikawa and HEC-1A were cultured in RPMI1640 (GIBCO-BRL) supplemented with 10% fetal bovine serum (FBS), 100 units/ml streptomycin and penicillin at 37 °C in a humidified atmosphere with 5% CO_2_.

### Cell proliferation assay

3000 cells were plated onto 96-well plates in triplicate and cultured overnight. Then, cells were treated with gradient concentration (0–15 μmol/L) of Pentamidine for indicated time. The one solution cell proliferation assay (MTS assay) (Promega) was used to measure growth viability of cells.

### Colony formation assay

Three thousands cells were added to each well of 6-well plates and incubated overnight. After cultured with different concentrations of pentamidine for 10 days, the cells were washed 3 times with PBS and fixed with 4% paraformaldehyde for 30 min. Then the cell colonies were stained with crystal violet and followed by counting with the ImageJ software.

### Cell Cycle analysis

Cells were cultured with vehicle or 15 μmol/L pentamidine in 6-well plates for 24 h. After culture, cells were harvested, washed with phosphate buffer saline (PBS), and then suspended in propidium iodide/ RNase solution (BD Biosciences, San Jose, CA, USA). Finally, the cells were analyzed using a Becton–Dickinson FACS Vantage flow cytometer (Becton Dickinson, San Jose, CA, USA).

### Wound healing assay

The cells were seeded in 6-well culture plates and cultured until reaching confluence. The wound was made using a sterile plastic pipette tip and the wells were washed with PBS to remove debris. Then, the cells were treated with Pentamidine or vehicle for indicated time in RPMI1640 medium containing 1% FBS. Scratches were observed and photographed under a light microscope at 0 h, 24 h, 48 h and 72 h after scratching. The relative migration of cells was quantified by the ImageJ software.

### Transwell assay

3 × 10^4^ cells in 100 μL serum-free medium were placed in upper chambers of 24-well cell culture plates with 8-μm pore filter inserts (Corning) and 600 μL medium with 10% FBS was added in the lower chamber. Pentamidine or vehicle was added into both upper chambers and lower chambers. After 24–36 h, cells on the upper surface were removed with cotton swabs and cells on the lower surface were fixed with 95% ethanol and stained with crystal violet solution. Then the stained cells were photographed under a microscope and counted by ImageJ software.

### Cell transfection

HA-AKT expression plasmid was obtained from Sino Biological. The constitutively active AKT tagged with HA (CA-AKT) was constructed by Sino Biological. Transfections were performed according to manufacturer’s instructions of jetPRIME reagents (Polyplus-transfection).

### Western blot

CellS were collected upon Pentamidine treatment, and lysed using ice-cold RIPA buffer containing protease cocktail inhibitor (APExBIO). The extracted cellular protein were denatured with 6 × loading at 100 °C for 5 min, and separated by electrophoresis on SDS–polyacrylamide gel electrophoresis (PAGE) and electrotransferred to PVDF membrane. The membrane was blocked with 5% nonfat milk powder in PBST for 1 h at room temperature. Primary antibodies were anti-Phospho-AKT (Ser473) and anti-AKT (Cell Signaling Technology, CST), and anti-MMP-2, anti-MMP-9, anti- GAPDH and anti-HA tag (Proteintech). Then the membrane was washed with PBST for 3 times and incubated with secondary antibidies for 1 h at room temperature. The specific bands were visualized using ECL reagent (Thermo, MA, USA). Additionally, for Fig. 3F (the blot of HA), the blot was cut prior to hybridisation with antibodies during blotting. All original images were displayed in Additional file 1.

### Statistical analysis

All data were obtained from at least three independent experiments. The Graphpad 5.0 software was used for statistical analysis. The results were compared using one-way ANOVA. The results were presented as mean ± standard deviation, and a difference of *P* < 0.05 was deemed as statistically significant.

## Results

### Pentamidine suppresses the growth and proliferation of ECa cells

**Fig. 1:**
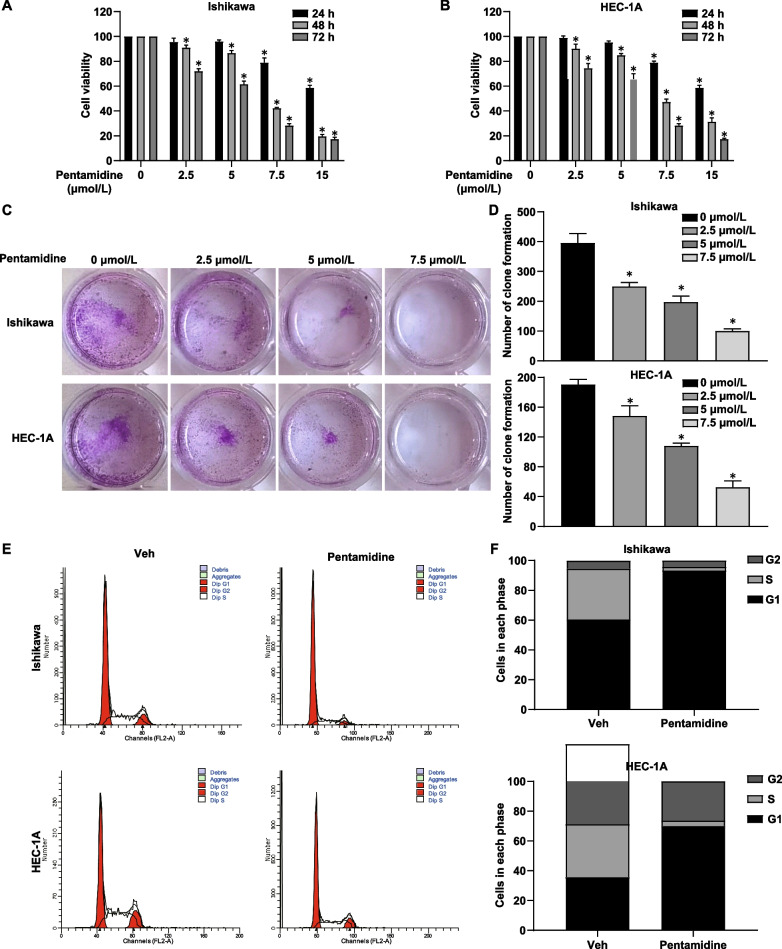
Pentamidine inhibits proliferation of Ishikawa and HEC-1A cells. **A**, **B** Ishikawa and HEC-1A cells were treated with Pentamidine (0, 2.5, 5.0, 7.5, 15 μmol/L) for 0, 24, 48 or 72 h. And then cell viability was determined by MTS assay. Statistical analysis was performed between cells treated with 0 μmol/L Pentamidine and each of the other drug concentration groups. Data are presented as mean ± standard deviation from three independent experiments. *P* value refers to one-way ANOVA. *P < 0.05. **C** Long-term colony formation assays of Ishikawa and HEC-1A cells induced by pentamidine (0, 2.5, 5.0, 7.5 μmol/L). **D** The number of colonies was calculated using ImageJ. Data are presented as mean ± standard deviation from three independent experiments. *P* value refers to one-way ANOVA. *P < 0.05. **E** Cell cycle distribution of Ishikawa and HEC-1A cells incubated with 5 μmol/L pentamidine for 24 h. **F** The percentage of cells at different cell cycle phases

Pentamidine has been reported to exhibit anticancer properties [11], therefore we investigated the effects of Pentamidine on EC. We first evaluated the effect of Pentamidine treatment on cell proliferation by MTS assays in EC cells. Pentamidine inhibited the proliferation of Ishikawa and HEC-1A cells in a dose-dependent manner, with maximum suppression observed at 15 μmol/L (Fig. 1A, B). In addition, Pentamidine was found to markedly suppress the ability of both Ishikawa and HEC-1A cells to form colonies in a dose-dependent manner (Fig. 1C, D).

To explore whether Pentamidine caused the arrest of cell cycle, we analyzed DNA content of two EC cell lines using flow cytometry analysis. PI staining was performed after treatment with an effective concentration of Pentamidine for 24 h in Ishikawa and HEC-1A cells. The results showed that Pentamidine induced an increase in the percentage of G1 phase cells along with a loss of cells in S phases, which intimated a block in G1/S progression (Fig. 1E, F). Taken together, these results suggest that Pentamidine has an anti-proliferative effect on EC cells.

### Pentamidine inhibits the migration and invasion of EC cells

**Fig. 2:**
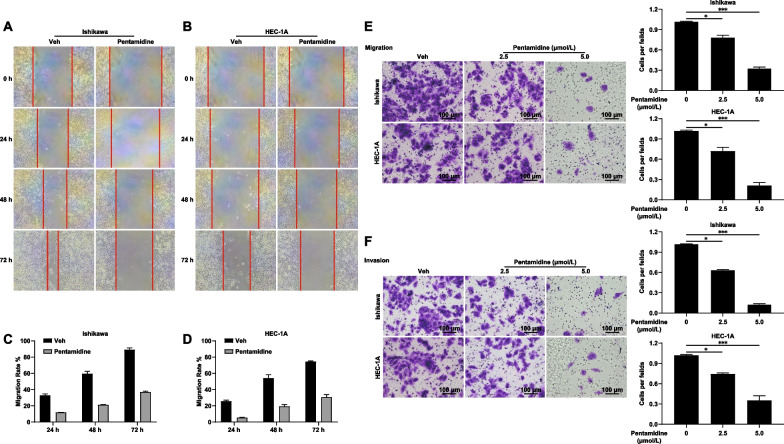
Pentamidine suppresses the migration and invasion of Ishikawa and HEC-1A cells. **A**, **B** Ishikawa and HEC-1A cells were treated with DMSO or Pentamidine for the indicated time, cell migration capacity was detected by wound-healing assay. Representative images were shown. **C**, **D** Quantitative analysis of wounding-healing assay. Data are presented as mean ± standard deviation from three independent experiments. **E** Ishikawa and HEC-1A cells were treated with the indicated concentrations Pentamidine for 24–36 h, the migration capacity were detected by Transwell migration assay. Representative images were photographed. Scar bar: 100 μm. Data are represented as mean ± standard deviation from three independent experiments. *P < 0.05; **P < 0.01. **F** Ishikawa and HEC-1A cells were treated with Pentamidine for 24–36 h, the cell invasion ability were detected by Transwell invasion assay. Representative images were photographed. Scar bar: 100 μm. Data are represented as mean ± standard deviation from three independent experiments. *P < 0.05; **P < 0.01; ***P < 0.001

WE next investigated whether Pentamidine affected the migration abilities of Ishikawa and HEC-1A cells by wound healing assays and Transwell migration assays. The results demonstrated that the migration rate of Ishikawa cells and HEC-1A cells decreased by 52.39% (Fig. 2A, B) and 44.43% (Fig. 2C, D) respectively after 72 h of treatment compared with the control group. Similarly, transwell migration assays without Matrigel results also illustrated that cell migration was reduced by Pentamidine in a dose- and time-dependent manner (Fig. 2E).

In Transwell invasion assays with Matrigel, both Ishikawa and HEC-1A cells treated with Pentamidine (2.5 μmol/L and 5 μmol/L) displayed much lower invasion ability than the control cells (Fig. 2F). These results suggest that Pentamidine represses the migration and invasion of EC cells.

### Effects of Pentamidine on PI3K/AKT pathway and the expression of MMPs proteins

**Fig. 3:**
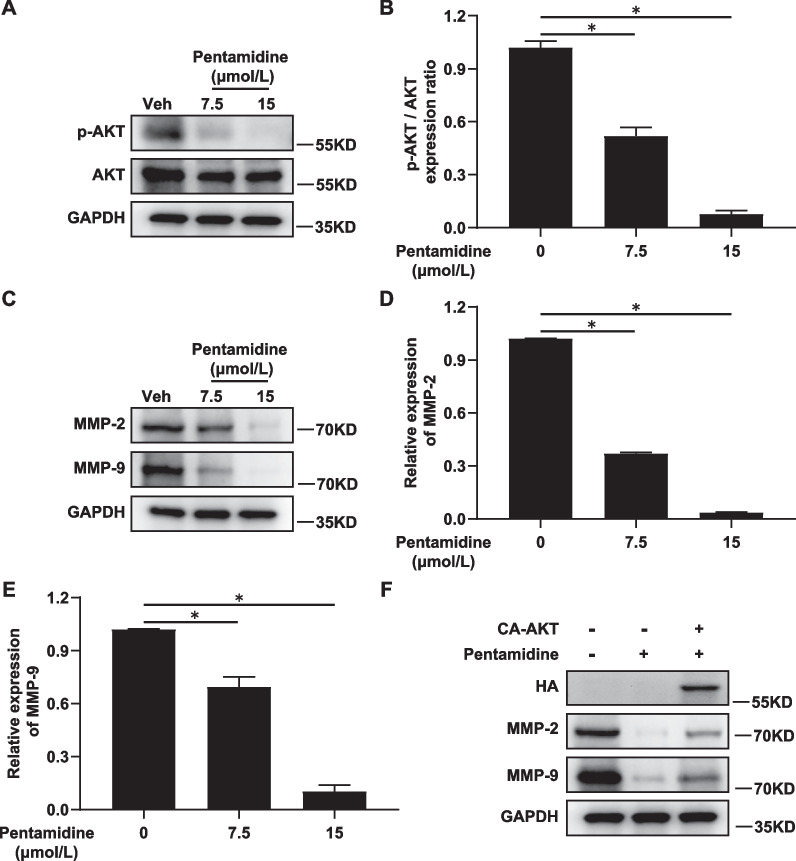
The effect of Pentamidine on the expression of p-AKT, MMP-2 and MMP-9 proteins in EC cells. **A** Ishikawa cells were treated with or without indicated concentrations of Pentamidine for 24 h. Protein levels of AKT and p-AKT were analyzed by Western blotting. GAPDH was used as the loading control. **B** Densitometric quantification of Western blot data presented in panel **A**. Data are represented as mean ± SD from three independent experiments. *P < 0.05. **C** Ishikawa cells were treated with or without indicated concentrations of Pentamidine for 24 h. Protein levels of MMP-2 and MMP-9 were analyzed by Western blotting. GAPDH was used as the loading control. **D**, **E** Densitometric quantification of Western blot data presented in panel **C**. Data are represented as mean ± standard deviation from three independent experiments. *P < 0.05. GAPDH was used as the loading control. **F** Ishikawa cells were respectively transfected with empty vector and HA-CA-AKT construct, and then treated with Pentamidine (15 μmol/L) or vehicle for 24 h. Protein levels of HA-tag, MMP-2 and MMP-9 were analyzed by Western blotting. GAPDH was used as the loading control. The blot (HA) was cut prior to hybridisation with antibodies during blotting

Given the central importance of PI3K/AKT pathway in EC carcinogenesis, we next tested whether Pentamidine has effects on this pathway. As shown in Fig. 3A, B and Additional 1: Figure S1, the phosphorylated and total level of AKT was detected by Western blotting, and the data showed that the protein level of p-AKT decreased in a dose-dependent manner, while the level of total AKT did not change significantly. The results suggest that Pentamidine is involved in suppressing the phosphorylation and activation of AKT.

Matrix metallopeptidases (MMPs), including matrix metallopeptidase-2(MMP-2) and -9 (MMP-9), can promote cells to migration through the basement membrane [25]. In order to confirm whether Pentamidine-induced suppression of migration and invasion in EC cells is associated with MMPs, the levels of MMP-2 and MMP-9 proteins were measured by Western blotting in Ishikawa cells. The results showed a decrease in MMP-2 and MMP-9 levels after treated with Pentamidine (Fig. 3C–E and Additional file 1: Figure S2). Then, Ishikawa cells were transfected with mammalian expression vectors expressing constitutively active p-AKT (CA-AKT) and empty vector respectively, and the corresponding changes in MMP-2 and MMP-9 expression were verified by Western blotting. As shown in Fig. 3F and Additional file 1: Figure S3, ectopic expression of CA-AKT reversed the pentamidine-induced decrease of MMP-2 and MMP-9, indicating Pentamidine inhibits MMP-2 and MMP-9 expression via PI3K/AKT pathway.

### Effects of combination of Pentamidine and LY294002 on cell proliferation in Ishikawa cells

**Fig. 4:**
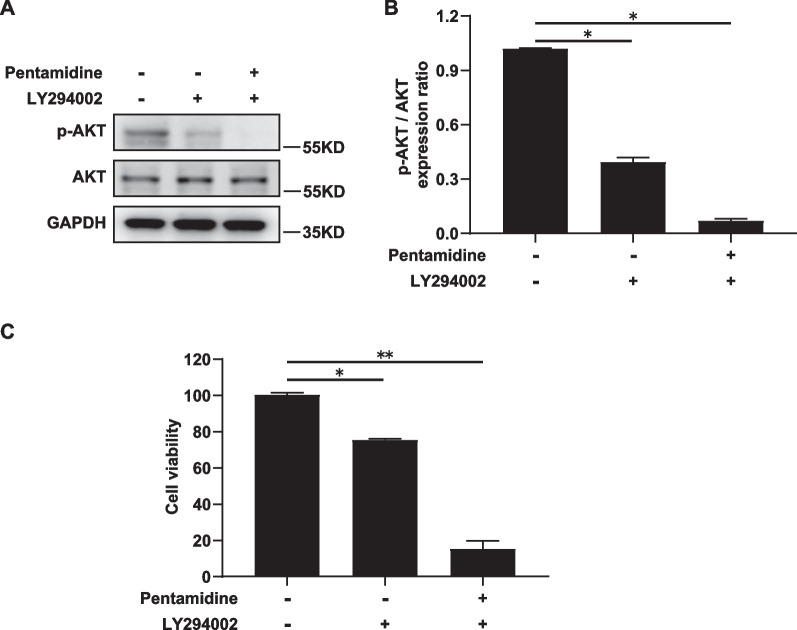
Effects of combination of Pentamidine and LY294002 on Ishikawa cell proliferation. **A** Ishikawa cells were treated with vehicle, LY294002 or LY294002 combined Pentamidine for 24 h. Protein levels of AKT and p-AKT were analyzed by Western blotting. GAPDH was used as the loading control. **B** The ratio of p-AKT/AKT was quantified by densitometry. Data are represented as mean ± standard deviation from three independent experiments. *P < 0.05. GAPDH was used as the loading control. **C** Ishikawa cells were treated with vehicle, LY294002 or LY294002 combined Pentamidine for 24 h. And then cell viability was determined by MTS assay. Statistical analysis was performed between cells treated with vehicle and each of the other drug groups. Data are represented as mean ± standard deviation from three independent experiments. *P* value refers to one-way ANOVA. *P < 0.05; **P < 0.01

We further examined the influence of the combination of Pentamidine and LY294002 on PI3K/AKT pathway and cell proliferation. Ishikawa were treated with vehicle, LY294002 (20 μM) and LY294002 (20 μM) combined with Pentamidine (10 μmol/L), respectively, for 24 h, and then the total and the phosphorylation levels of AKT were detected by Western blotting. As shown in Fig. 4A, B and Additional file 1: Figure S4, the level of p-AKT was reduced approximately fourfold and tenfold by treatment with a combination of LY294002 and Pentamidine compared to treatment with LY294002 alone or vehicle, respectively. However, we have not detected the obvious change of the total protein level of AKT. This data suggests that the combination of LY294002 and Pentamidine results in potent inactivation of PI3K/AKT pathway. Meanwhile, co-treatment with LY294002 and Pentamidine showed a significantly greater inhibitory effect on Ishikawa cell viability than treatment with LY294002 alone or vehicle (Fig. 4C). Our results demonstrate that Pentamidine enhances LY294002-induced repression of proliferation in Ishikawa cells.

## Discussion

In this study, we provided evidence to demonstrate that Pentamidine exerts a profound inhibitory effect on EC progression, as attested by reduced proliferation, migration and invasion. Our results showed that Pentamidine could be used in potential therapeutic strategies for EC by targeting PI3K/AKT/MMP-2/9 signaling pathway.

PI3K/AKT is an essential signaling pathway and plays a crucial role in the progression of cancers [22]. Particularly, more than 80% of cases in endometrial cancer harbor at least one somatic variation that influences signal pathways, and the PI3K/AKT signaling pathway is one of the most frequently changed protein signal transduction pathways in EC [22]. EC has more frequent mutations in the PI3K/AKT pathway than any other tumor type studied by The Cancer Genome Atlas [8]. Activation of the PI3K/AKT pathway is considered a marker of cancer progression, including that in EC [26–28]. It has been reported that the overexpression of Erb-B2 Receptor Tyrosine Kinase 2 (ERBB2), mutations of EGFR/PI3K pathway, or the loss of Phosphatase and Tensin Homolog (PTEN), as well as mutations or amplification of AKT itself can result in increased PI3K-AKT signaling in tumor cells [26, 29]. Mutation or deletion of PTEN is common in many kinds of cancers and results in overactivation of the PI3K/AKT network. Restoration of PTEN function enhances p21^WAF1/CIP1^-regulated cell-cycle inhibition by blocking PI3K/ AKT signal pathway [26, 30]. Moreover, mutations in PTEN gene are present in up to 70% of type1 and 35% of type 2 ECs [27]. Mutations in PIK3CA lead to increased activation of the PI3K/AKT/mTOR pathway, occurring in 41–52% of type 1 and 33% to 38% of type 2 ECs [27]. These data hinted that PI3K/AKT signal pathway plays a vital role in EC.

The incidence of EC is increasing and yet survival has not improved substantially over the past 30 years [27]. Although most cases are detected and treated at an early stage, a significant number of women present at an advanced or recurrent stage and have a poor prognosis [27, 31]. Treatment outcomes of patients with advanced or recurrent EC are still unsatisfactory [27]. Currently, treatment outcomes may be potentially improved with PI3K/AKT pathway inhibitor alone or combination therapy with other drugs such as anti-endocrine drugs, for a series of solid tumors, including endometrial cancer [27]. Hence, the prognosis of women with advanced or recurrent EC may be significantly improved by therapy targeting PI3K/AKT pathway. Our data showed that Pentamidine treatment markedly suppresses proliferation, migration and invasion (Figs. 1, 2) and decreases p-AKT levels in dose-dependent manner (Fig. 3A, B). Moreover, a combination of Pentamidine and LY294002 result in increased inhibition of cell proliferation and downregulation of p-AKT levels (Fig. 4). These results suggested that Pentamidine may serve as a potential inhibitor of PI3K/AKT signaling pathway in EC.

Invasion and metastasis are critical steps in cancer progression [25]. MMPs play remarkable roles in migration, metastasis and invasion of tumor cell [25]. Through multiple molecular mechanisms, the invasiveness and metastasis of tumors can also be directly potentiated by overactivation of P13K/AKT pathway. Meanwhile, activated PI3K/AKT pathway promotes the degradation of extracellular matrix by upregulating the expression of MMPs, which contributes to the invasion and metastasis of cancer cells [32, 33]. In the present study, we found that Pentamidine treatment leads to a significant decrease in the level of MMP-2 and MMP-9 expression via PI3K/AKT signaling pathway (Fig. 3C–F), suggesting that Pentamidine has the potential to inhibit migration and invasion through P13K/AKT/MMP-2/9 pathway in EC.

## Conclusions

In summary, we demonstrated that Pentamidine, a cationic drug, can inhibit cell proliferation, migration and invasion in Ishikawa and HEC-1A cells, and these effects might be associated with the suppression of P13K/AKT/MMP-2/9 pathway. These results provide a novel perspective for the possible use of Pentamidine in chemotherapeutic interventions and a combinational therapy with other approaches for the treatment of EC. However, in the future, the clinical application of Pentamidine needs to be further evaluated and further experiments are needed to illustrate the mechanism of involvement of Pentamidine in inhibition of PI3K/AKT signaling pathway.

## Supplementary Information

**Additional file 1.** All original and unprocessed images of Western blots.

## Data Availability

The data that support the findings of this study are available from the corresponding author upon reasonable request.

## Abbreviations

EC: Endometrial cancer
OS: Overall survival
HIF-1a: Hypoxia-inducible factor 1
ATCC: American Type Culture Collection
MMPs: Matrix metallopeptidases
