# Supplementary material for: Pentamidine inhibits proliferation, migration and invasion in endometrial cancer via the PI3K/AKT signaling pathway
Source: BMC Womens Health. 2022 Nov 24;22:470. doi: 10.1186/s12905-022-02078-1 (PMC9700983; doi:10.1186/s12905-022-02078-1)
Supplement: Supplementary file 1 — Additional file 1. All original and unprocessed images of Western blots. [file 12905_2022_2078_MOESM1_ESM.pdf]

Supplementary figure 1

figure 3A

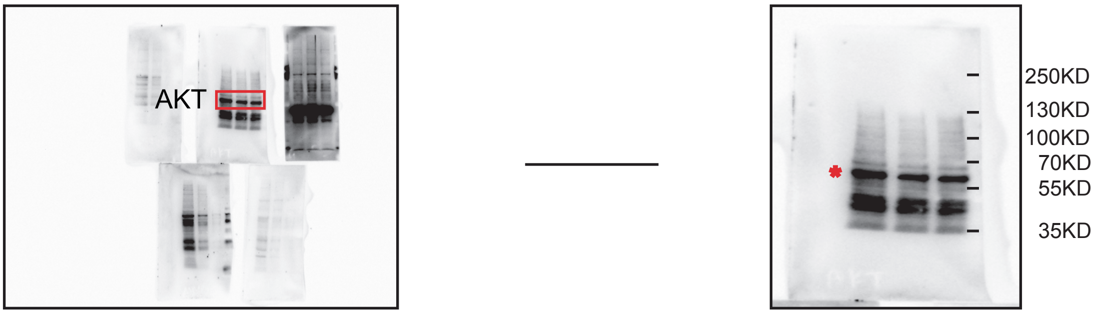

AKT

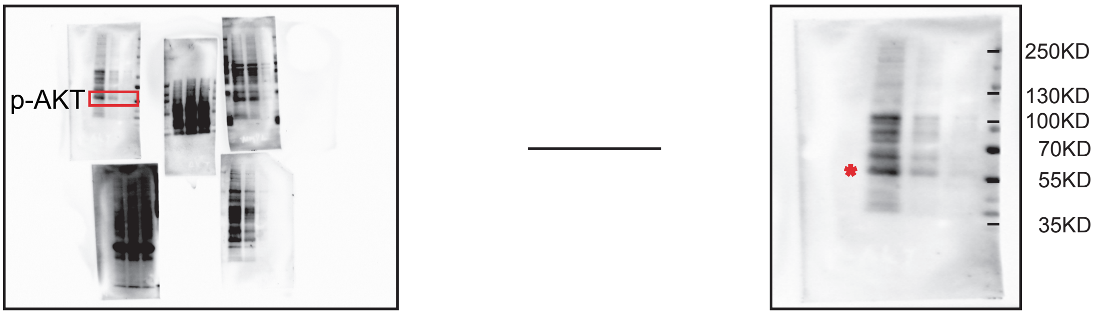

p-AKT

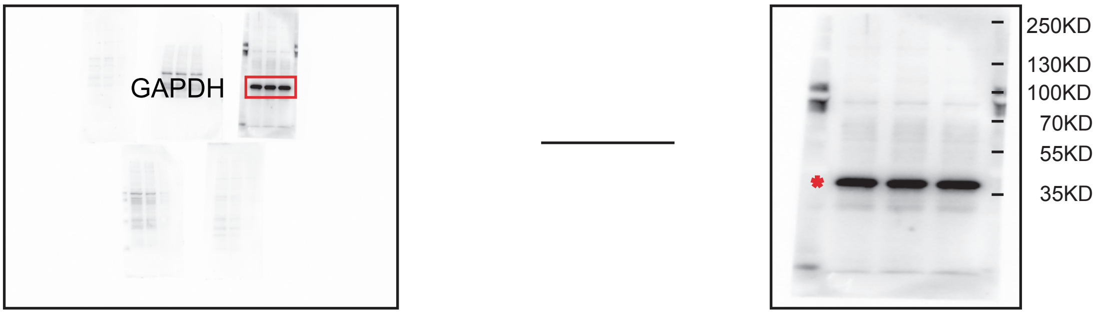

GAPDH

Supplementary figure 2

figure 3C

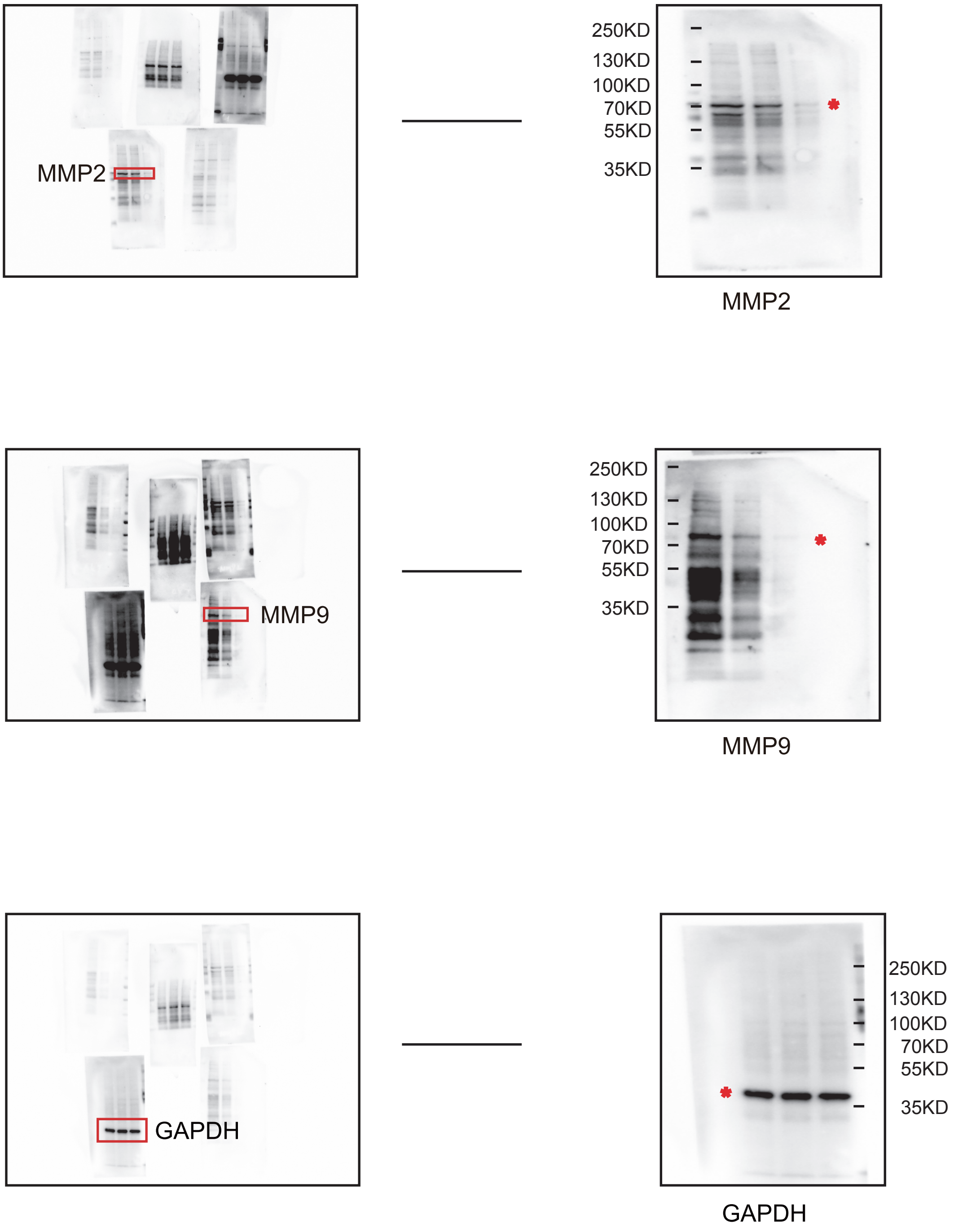

Supplementary figure 3

figure 3F

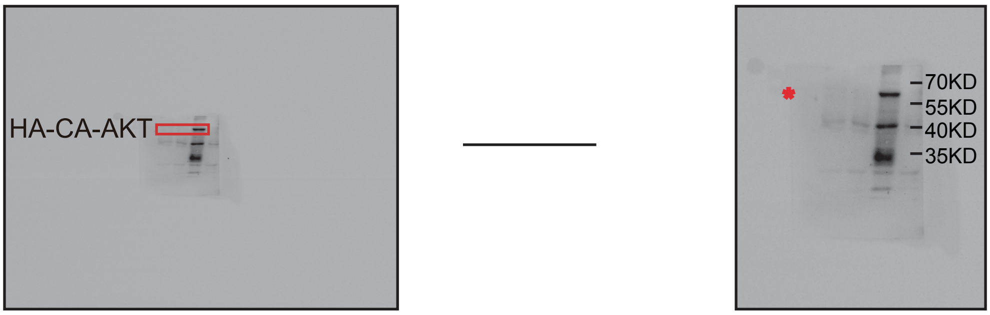

HA-CA-AKT

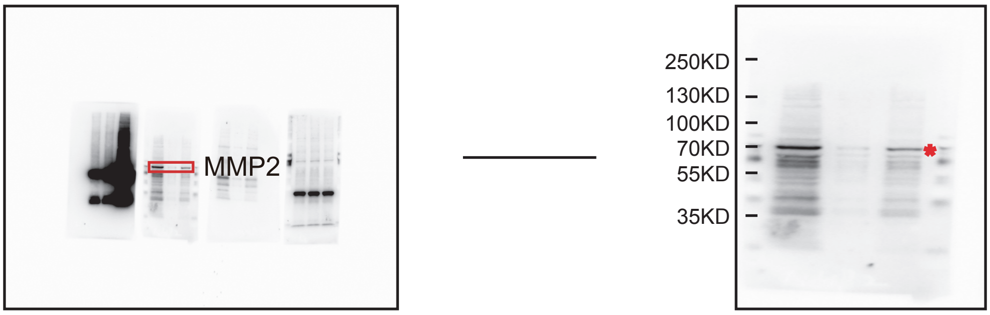

MMP2

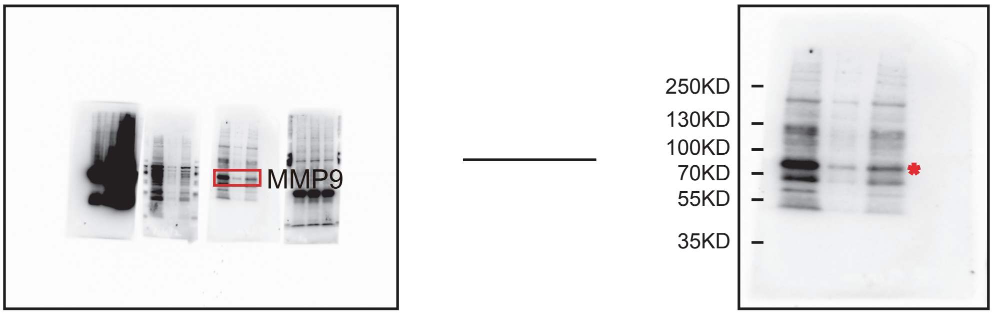

MMP9

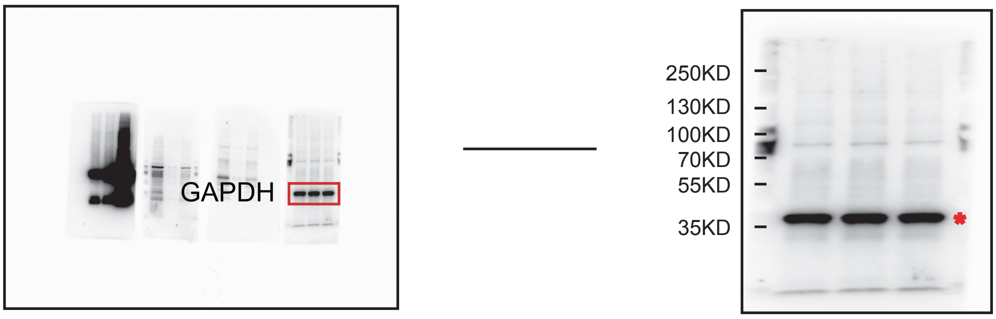

GAPDH

Supplementary figure 4

figure 4A

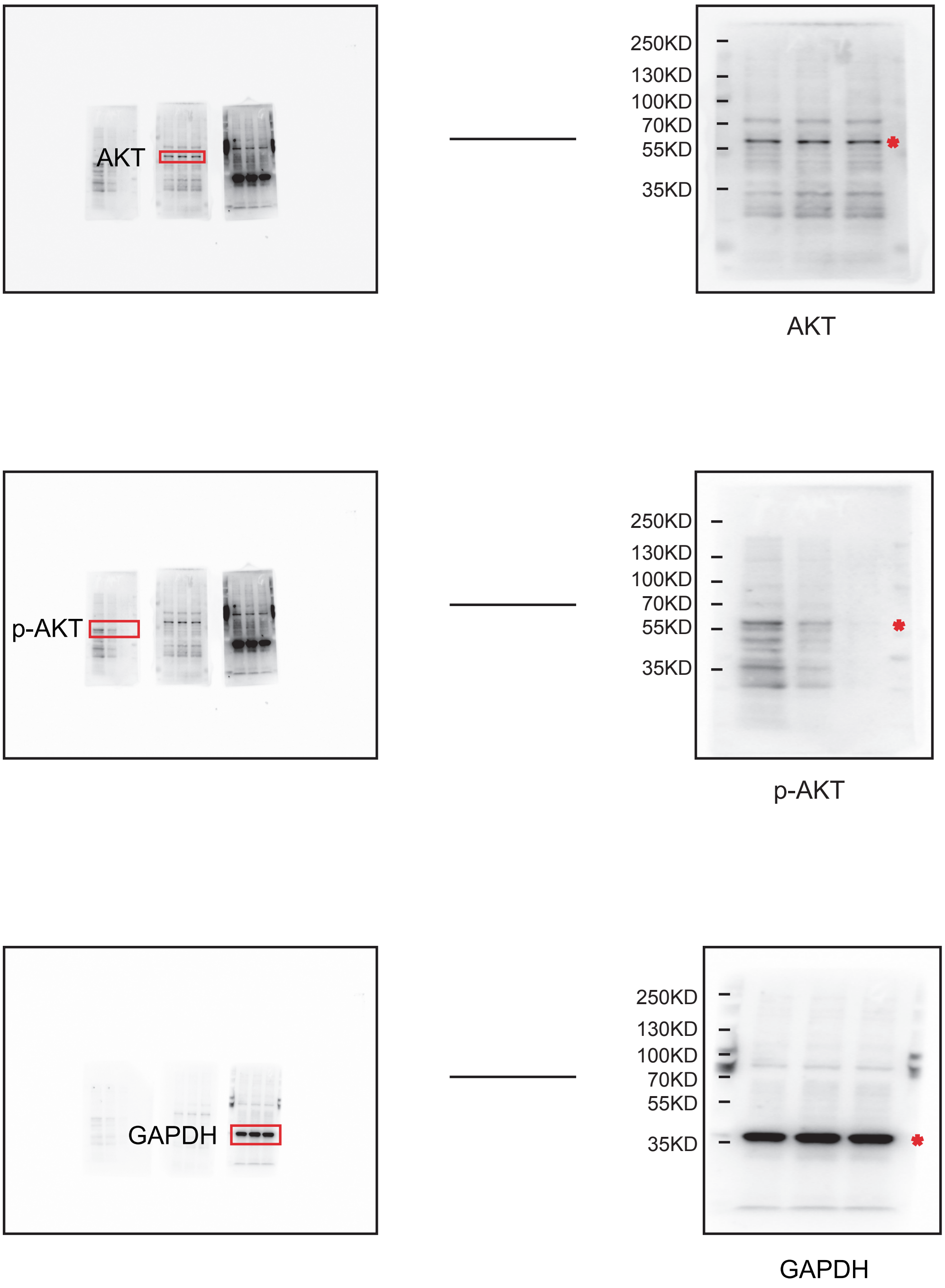

Supplementary figure legends

Supplementary figure 1

The treatment of Pentamidine reduces the expression of p-AKT protein in Ishikawa cells.

Supplementary figure 2

The treatment of Pentamidine decreases the expression of MMP-2 and MMP-9 protein in Ishikawa cells.

Supplementary figure 3

Ectopic expression of HA-CA-AKT reversed the pentamidine-induced decrease of MMP-2 and MMP-9 in Ishikawa cells.

Supplementary figure 4

The treatment with a combination of LY294002 and Pentamidine significantly decreases the expression of p-AKT in Ishikawa cells.
